# Developing a translational research framework for MDD: combining biomolecular mechanisms with a spiraling risk factor model

**DOI:** 10.3389/fpsyt.2024.1463929

**Published:** 2025-01-07

**Authors:** Max van Baalen, Lars van der Velden, Toon van der Gronde, Toine Pieters

**Affiliations:** ^1^ Department of Pharmaceutical Sciences and Freudenthal Institute, Utrecht University, Utrecht, Netherlands; ^2^ Late-Stage Development, Oncology Research and Development, AstraZeneca, New York, NY, United States

**Keywords:** MDD (major depressive disorder), microbiota-gut-brain axis, HPA axis, pro-inflammatory state, translational research framework, dysbiosis, Biopsychosocial approach, spiraling risk factor model

## Abstract

**Objective:**

The global incidence and burden of Major Depressive Disorder (MDD) are increasing annually, with current antidepressant treatments proving ineffective for 30-40% of patients. Biomolecular mechanisms within the microbiota-gut-brain axis (MGBA) may significantly contribute to MDD, potentially paving the way for novel treatment approaches. However, integrating the MGBA with the psychological and environmental aspects of MDD remains challenging. This manuscript aims to: 1) investigate the underlying biomolecular mechanisms of MDD using a modeling approach, and 2) integrate this knowledge into a comprehensive ‘spiraling risk factor model’ to develop a biopsychosocial translational research framework for the prevention and treatment of MDD.

**Methods:**

For the first aim, a systematic review (PROSPERO registration) was conducted using PubMed, Embase, and Scopus to query literature published between 2016–2020, with select additional sources. A narrative review was performed for the second aim.

**Results:**

In addition to genetics and neurobiology, research consistently indicates that hyperactivation of the HPA axis and a pro-inflammatory state are interrelated components of the MGBA and likely underlying mechanisms of MDD. Dysregulation of the MGBA, along with imbalances in mental and physical conditions, lifestyle factors, and pre-existing treatments, can trigger a downward spiral of stress and anxiety, potentially leading to MDD.

**Conclusions:**

MDD is not solely a brain disorder but a heterogeneous condition involving biomolecular, psychological, and environmental risk factors. Future interdisciplinary research can utilize the integrated biopsychosocial insights from this manuscript to develop more effective lifestyle-focused multimodal treatment interventions, enhance diagnosis, and stimulate early-stage prevention of MDD.

**Systematic Review Registration:**

https://www.crd.york.ac.uk/PROSPERO/, identifier CRD42020215412.

## 1 Introduction

Major Depressive Disorder (MDD) is a prevalent mental health condition affecting approximately 280 million people worldwide, which accounts for about 5% of the adult population (1). Characterized by persistent depressed mood most of the day, nearly every day for at least two weeks, depressive episodes are often accompanied by symptoms such as disrupted appetite and sleep, poor concentration, and feelings of excessively low self-worth. These symptoms significantly impact quality of life and can lead to severe consequences, including an increased risk of suicide, with individuals suffering from MDD being nearly twenty times more likely to commit suicide compared to those without depression (2).

MDD is a heterogeneous disorder with complex origins, including varied genetic and environmental factors (3, 4). Approximately 35%–40% of depression cases are inherited, suggesting that external factors such as adverse life experiences account for the remaining 60%–65% (5, 6). Risk factors for MDD include childhood trauma, substance use disorders, and low socioeconomic status (7).

Current treatments for MDD encompass psychotherapy (e.g., cognitive behavioral therapy, supportive therapy, and psychoeducation), pharmacological treatments (e.g., selective serotonin reuptake inhibitors (SSRIs), tricyclic antidepressants (TCAs), and ketamine), and somatic treatments (e.g., electroconvulsive therapy) (7, 8). Although these interventions are effective for some patients, the Global Burden of Disease Study 2019 indicates that the incidence and burden of depression among young people (ages 10-24) have been increasing annually over the past decade (9). This trend raises questions about the efficacy of the serotonin hypothesis, which has dominated MDD research and treatment for decades, and the widespread use of SSRIs (10).

For 30-40% of patients, antidepressants do not provide adequate responses (3, 5). Moreover, one-third of MDD patients show no response even after four lines of antidepressant treatment (5, 11). A recent systematic review found no consistent evidence linking serotonin with depression, further challenging the serotonin hypothesis (10). Additionally, withdrawal symptoms upon discontinuing antidepressants present another significant issue (12, 13). These findings underscore the urgent need to deepen our understanding of the dynamic and complex mechanisms underlying MDD.

This manuscript aims to: 1) investigate the underlying biomolecular mechanisms of MDD using a modeling approach, and 2) integrate this knowledge with a new comprehensive ‘spiraling risk factor model’ to inform integrated biopsychosocial treatment approaches for MDD. The research question guiding this work is: *How can the biomolecular mechanisms underlying MDD be related to psychological and environmental risk factors, and how can they be integrated into a translational research framework?*

Recent research suggests that MDD is more complex than a brain-only disease, involving a dysregulated hypothalamic-pituitary-adrenal (HPA) axis, a pro-inflammatory state, and dysbiosis of the microbiota-gut-brain axis (MGBA) (7). These complex mechanisms are mediated by key biomolecules such as cytokines enhancing the pro-inflammatory state like interleukin-6 (IL-6) and tumor necrosis factor (TNF) (14), short-chain fatty acids (SCFAs) (15, 16), cortisol (17), brain-derived neurotrophic factor (BDNF) (18), neurotransmitters (NTs) (19, 20), and lipopolysaccharide (LPS) (21). While fundamental knowledge about these underlying biomolecular mechanisms exists, a comprehensive picture of their interrelations is lacking. Therefore, we propose a schematic model incorporating the MGBA, HPA axis, and immune system in relation to MDD (**Figure 2**).

Moreover, MDD results from a combination of biomolecular, environmental, and psychological risk factors (7). Dysbiosis in the MGBA, imbalances in mental and physical conditions, lifestyle factors, and pre-existing treatments all contribute to the disorder. For example, van der Gronde et al. (5) describe how chronic stress and failure to cope can trigger a downward spiral of stress and anxiety, potentially leading to MDD. Thus, we aim to combine the available knowledge of the biomolecular mechanisms of MDD with psychological and environmental risk factors into a comprehensive spiraling risk factor model (**Figure 3**). This biopsychosocial model is intended to develop a translational research framework for the prevention and treatment of MDD.

## 2 Methods

For our first aim, we carried out a systematic review that focused on research data concerning the role of the MGBA in the etiology of MDD, described in the sections 4 and 5 of this manuscript. The databases used were EMBASE, PubMed and Scopus, and the review has been pre-registered at PROSPERO under registration number CRD42020215412.

Studies were included that had a focus on the effects of stress, inflammation, microbiota, the gut-brain axis, external influences and depression.

Clinical trials and studies, as well as other research findings have been gathered through PubMed, Scopus and EMBASE using the following search strings:

- EMBASE: (‘depression’/exp OR ‘stress’/exp) AND (‘microflora’/exp OR ‘microbiome’/exp) AND ‘inflammation’/exp AND (2016:py OR 2017:py OR 2018:py OR 2019:py OR 2020:py) AND (‘article’/it OR ‘conference abstract’/it OR ‘review’/it).
- PubMed (limited from 2016-2020): (“depressive disorder”[All Fields] OR “stress”[All Fields]) AND (“microbiota”[All Fields] OR “gastrointestinal microbiome”[All Fields]) AND “inflammation”[All Fields] based on MeSH terms.
- Scopus: TITLE-ABS-KEY ((“depression” OR “stress”) AND (“microflora” OR “microbiome”) AND “inflammation”) AND (LIMIT-TO (PUBYEAR, 2020) OR LIMIT-TO (PUBYEAR, 2019) OR LIMIT-TO (PUBYEAR, 2018) OR LIMIT-TO (PUBYEAR, 2017) OR LIMIT-TO (PUBYEAR, 2016)).

The search strings were based on keywords relating to the research question, building on searching strategies of prior research. The keywords that had been found were (any derivations of) inflammation, stress, depressive disorder, gastrointestinal biome.

Only peer-reviewed studies written in English from 01 January 2016 until 14 August 2020 were included. The systematic review does not include translated studies, book chapters, conference abstracts, methodology reports and editorials.

Articles from the search were included by LV based on title and abstract and finally on full-text assessment. Judgements made regarding the inclusion of articles were carefully supervised by TP. Excluded articles and their specific exclusion rationality can be found in **Supplementary Table 1**. Risk of bias assessment was performed by LV and MB for the initial database search and reviewed by TP. Risk of bias assessment for externally included studies was performed by both MB and LV and reviewed by TP. Assessment was done manually and no automation tools were used.

A wide variety of studies regarding different aspects relating to our research question were included. This was done to maximize different perspectives regarding the research question. This includes, for example, the role of immune cells in MDD, as the microbiota can interact with these immune cells. But also some studies on for instance inflammatory bowel disease, as there are phenotypic similarities to MDD. Studies that offer minimal to no insight on MDD were excluded (TP, LV, MB).

**Figure 1:**
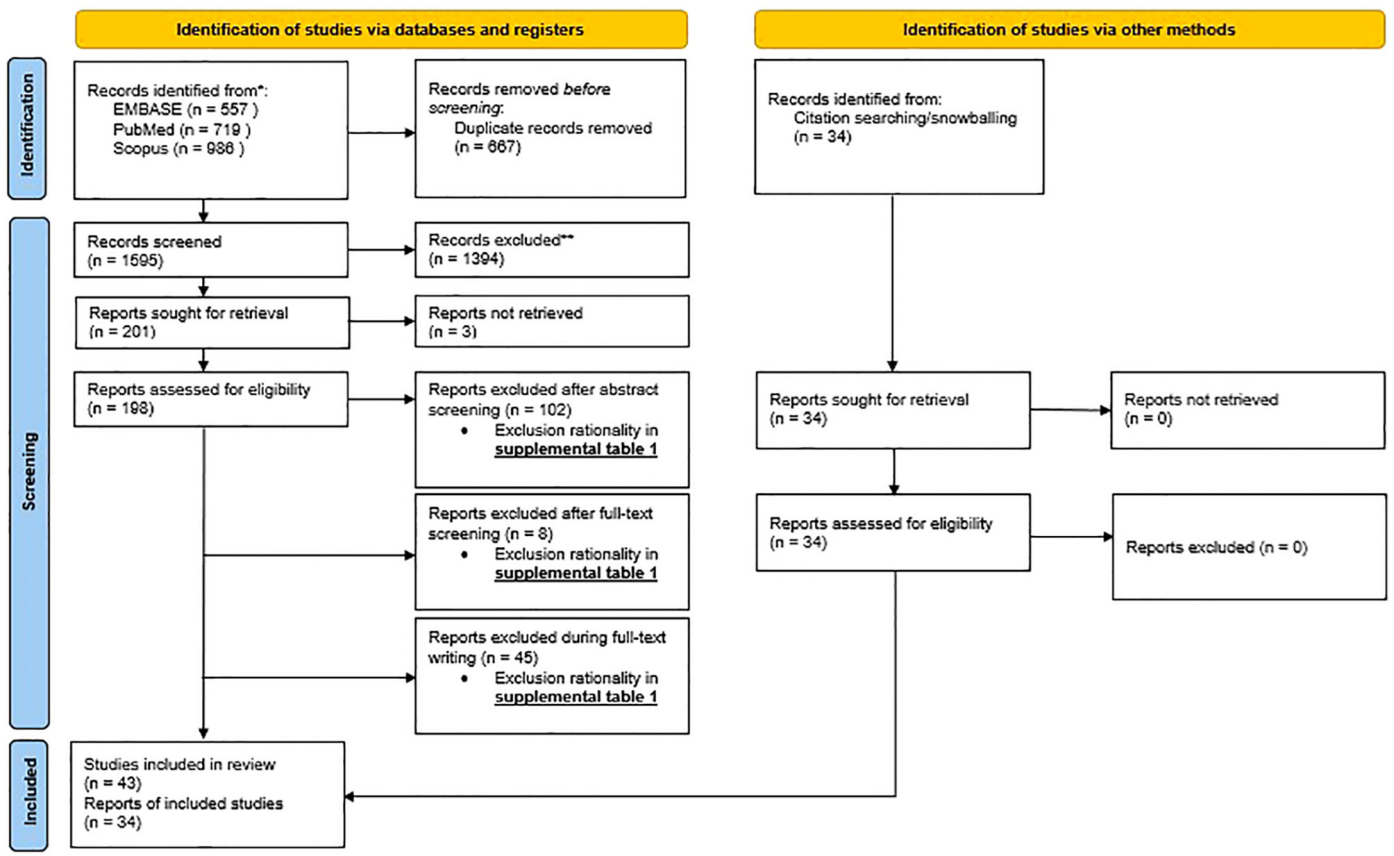
PRISMA flowchart: PRISMA 2020 flow diagram for new systematic review which included searches of databases, registers and other sources.

To further maximize the identification of eligible articles related to the research question, external studies were included through websites and citation searching/snowballing, according to the PRISMA 2020 protocol (**Figure 1**, PRISMA flowchart).

For our second aim, we integrated the results of our systematic review for the first aim in a narrative review, described in section 6. Mainly PubMed was used to search for relevant publications, with the preference for recent systematic reviews and meta-analyses. To maximize different perspectives, externally found literature together with select additions of recent findings based on collective suggestions of the authors were added (applies to all sections of this article).

## 3 Results

For our systematic review (aim 1; section 4 and 5), from the 2262 articles originally retrieved via databases and registers, only 43 articles were included in the systematic review (**Figure 1**, PRISMA flowchart). Prior to screening, 667 articles were removed due to duplication. During screening, 1394 records were excluded on title, another 3 records were excluded due to inability to retrieve the record, yielding 198 articles assessed for eligibility. After abstract screening, full-text screening, and during writing, an additional 102, 8 and 45 records were excluded respectively. The details of the reason for exclusion can be found in **Supplementary Table 1**.

Through other methods including external sources and snowballing, an additional 34 articles were added. This yields a total of 77 articles the systematic review was based on.

For our narrative review (aim 2; section 6) we included an additional 89 articles found across mainly PubMed. As this second part of the article is a narrative review and not a systematic review, there is no PRISMA flow diagram shown for this part.

## 4 Major depressive disorder and possible biological etiological mechanisms

To develop a translational research framework for prevention and treatment of MDD, we start with what is known about the biological underlying mechanisms. As MDD is highly heterogeneous and associated with many comorbidities, the biology is intricate and not related to a specific factor. Partly, MDD has been associated with complex genetics and neurobiology (22). As most MDD patients experience a lot of stress because of a wide variety of stressors, we will also focus on the stress mechanisms (5, 15). Closely related to stress, the immune system is another universal finding in MDD, making the immune system an important mechanism as well (19). Furthermore, the disruption of the gut microbiome called ‘dysbiosis’ is underlying both the stress and the immune system, making it interesting for us to further elaborate on the topic of dysbiosis in this review. The MGBA plays a major role in the complex interplay of these mechanisms (21). The genetics, neurobiology, stress response, immune system, MGBA and the interplay of these processes related to MDD will be discussed in the upcoming sections (**Table 1**).

**Table 1:** Overview of (potential) biological etiological mechanisms in MDD and their impact on the disorder.

Biological etiological mechanism	Role or potential significance in MDD
Genetics	- DRD2 (emotional processing) and CELF4 (synaptic activity) genes possibly involved (23) - Possible epigenetic pathways (e.g., histone acetylation, DNA methylation) related to MDD and risk factors (24) - Large amount of candidate genes because of heterogeneous character of MDD (22)
Neurobiology	- Debated association between MDD and neurotransmitters, such as serotonin (10, 25) - Disruption of frontoparietal network, salience network, and default mode network (7) - Impaired brain regions: prefrontal cortex, anterior cingulate, orbitofrontal cortex, insula, and hippocampus (26)
Stress	- Hyperactive HPA axis possibly triggered by exposure to (chronic) stressors (5, 30, 31, 35, 36) - Increased cortisol levels (32)
Immune system	- Increased levels of pro-inflammatory cytokines such as TNF, and IL-6 (14, 39) - Pro-inflammatory state (45) and systemic inflammation (46) - Positive feedback with HPA axis (15, 32, 43) - Increases permeability of BBB (19)
Gut microbiome	- Involved in HPA axis (30), immune system (50), BBB (19), and central nervous system (51) - Disruption of the GM / dysbiosis (49, 54)

### 4.1 Genetics

According to recent research, children of individuals with MDD face a 35–40% likelihood of experiencing MDD in early adulthood, which is twice the risk observed in offspring of parents without MDD (5, 7). This includes both genetic and environmental factors within the family, in which both factors roughly contribute equally.

Examples of genes that implicate a neurobiological etiology of MDD are the dopamine receptor D2 (*DRD2*) gene, which is related to emotion processing, and the CUGBP Elav-Like Family Member 4 (*CELF4*) gene, which is associated with regulating synaptic activity for excitatory neurons (**Table 1**) (23). Compared to the majority of other mental disorders, the heritability of depression of approximately 37% is relatively low. Although large (up to one million participants) genome-wide association studies (GWAS) identified 178 genetic risk loci and 200 candidate genes, the specificity and robustness of these results are questionable (22). This might be due to the fact that researchers adopted minimal phenotyping methodology to identify cases to obtain robust statistical significance. This comes with a cost, resulting in signals that are insufficiently attributable to MDD. Moreover, effect sizes of GWAS results are rather small (7). Moreover, epigenetic processes could play a role in facilitating interactions between genes and the environment. Researchers found some markers related to MDD and risk factors (such as childhood trauma) and epigenetic pathways such as histone deacetylases and DNA methyltransferases (24). However, the sample sizes were small and for some studies only animal research was performed.

The considerably weak results of genetics involved in MDD may partly be explained by the fact that it is a complex and heterogeneous disease. Moreover, the high number of comorbidities many patients experience may result in a wide variety of contributing factors that are not reducible to single genes or single nucleotide polymorphisms. Additionally, in terms of treatment applications, it is hard to develop genetic treatments to help patients with MDD. However, the robustness of genetics underlying MDD may increase in the future.

### 4.2 Neurobiology

Despite the inconsistent findings between serotonin and MDD, depression is still thought to be a disease in which the brain plays a crucial role (**Table 1**) (10). Moreover, the study by Moncrieff et al. (10) has raised significant critiques regarding its reliability, as highlighted in multiple correspondences available on their webpage. An important example is that they misinterpret some of the reviewed data and suggested that serotonin reuptake inhibitor antidepressants, such as SSRIs, may decrease rather than enhance serotonin function (25). Furthermore, MDD exhibits high heterogeneity and is more complex than simply attributing MDD to serotonin or excluding the role of serotonin completely.

Besides serotonin, MDD is associated with the disruption of networks and different brain regions (7). Functional magnetic resonance imaging (fMRI) studies reported certain hypo- and/or hyperconnectivities in three neural networks, namely, the frontoparietal network (higher order cognitive processes), the salience network (emotional and motivational stimuli) and the default mode network (self-referential thinking). Brain regions in the central nervous system (CNS) associated with MDD are the prefrontal cortex, anterior cingulate, orbitofrontal cortex, and insula (7). These brain regions play a role in emotional processing and cognitive control. For instance, a study analyzing MRI data from 10.105 people (of which 2148 were MDD patients) showed that grey matter density of the orbitofrontal cortex, anterior cingulate cortex, and insula was reduced in MDD patients compared to healthy controls (26). Furthermore, other research demonstrated that decreased postmortem hippocampal volume is associated with MDD (27). This aligns with data from MRI studies showing subtle increment of hippocampal volume in remitting MDD patients (28). However, structural brain differences in individuals with MDD exhibit small effect sizes, and are not specific to MDD (7). These kinds of structural differences can be found in other mental disorders like anxiety disorder as well (29). Moreover, the mechanisms underlying these structural changes are possibly alterations in dysregulation of the HPA axis, the immune system and the gut-brain axis. Together with the fact that a large proportion of patients do not find adequate relief from antidepressants that directly target brain function (such as SSRIs), it is worthwhile to explore these alternative biological mechanisms (5).

### 4.3 Stress

Hyperactivity of the HPA axis is found in many psychopathologies, including depression (30, 31). Symptoms of depression, such as disrupted sleep and hopelessness, have been associated with HPA axis impairments (**Table 1**). In humans, higher cortisol levels are found in more than 70% of MDD patients (32). Also in rats, research showed that an over-activated HPA axis increased anxiety and depressive-like behavior (15). Because of the significant relation between the HPA axis and depression, researchers believe that hyperactivity of the HPA axis is one of the most reliable biological markers of MDD (15, 33). High cortisol levels may therefore potentially function as a predictor for MDD onset (34). However, it is still not completely clear whether dysregulation of the HPA axis is a cause or consequence of depression.

**Figure 2:**
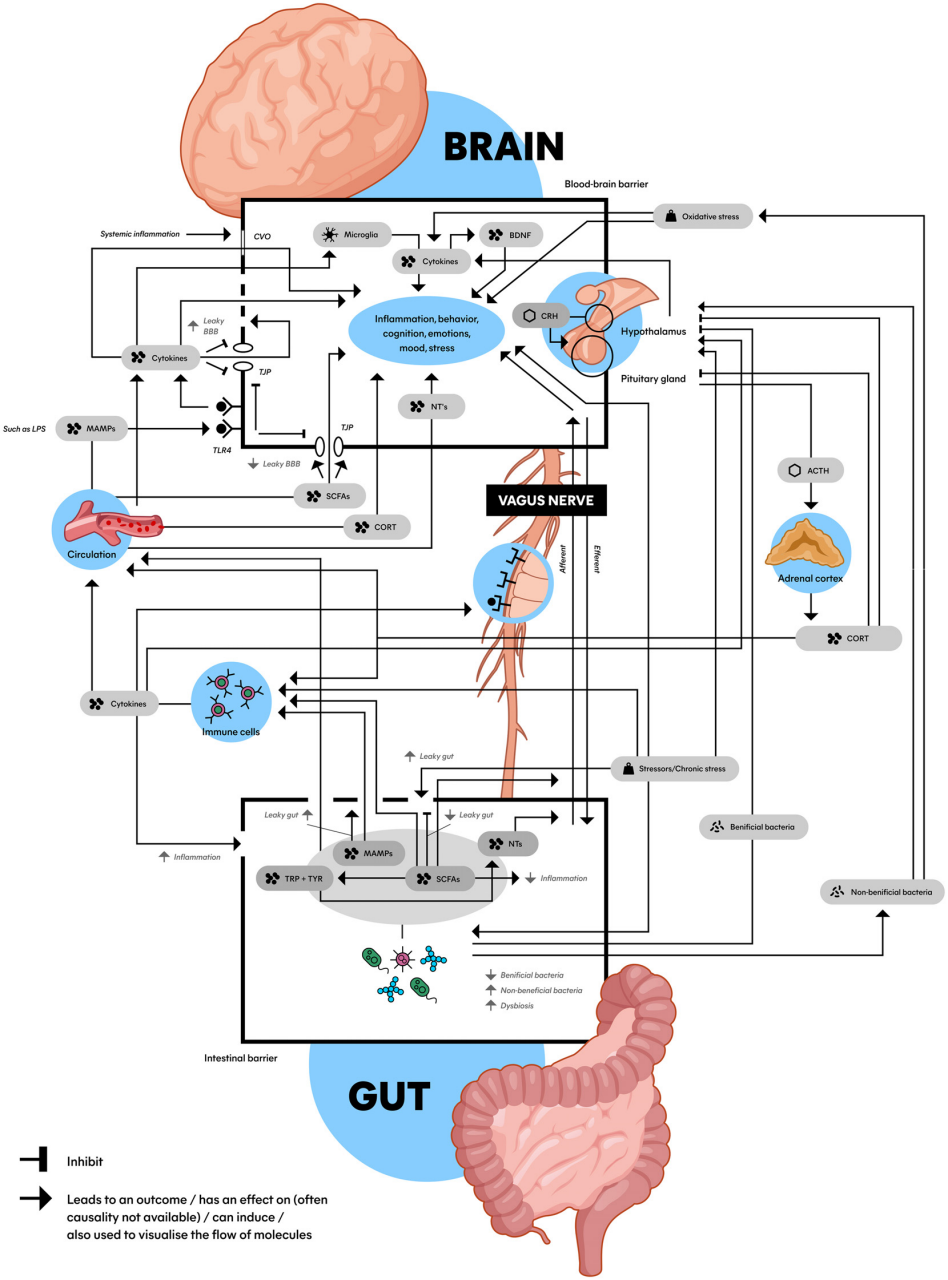
Schematic model of the microbiota gut-brain axis: An interplay between the HPA axis, the immune system, the gut microbiome and the brain connected by biomolecules. This figure shows the complex communication between the gut (bottom) and the brain (top) with a wide array of factors inducing, promoting, and inhibiting the stress (top right) and immune system (middle left) with an important role for the gut microbiota and microbial derivatives (bottom). Microbiota can be (indirectly) bidirectionally involved with a hyperactive HPA axis and pro-inflammatory state that play a role in the etiology of MDD. ACTH, adrenocorticotropic hormone; BBB, blood-brain barrier; BDNF, brain-derived neurotrophic factor; CORT, cortisol; CRH, corticotropin-releasing hormone; CVO, circumventricular organs; LPS, lipopolysaccharide; MAMPs, microbe-associated molecular patterns; NTs, neurotransmitters (serotonin, dopamine and norepinephrine); SCFAs, short-chain fatty acids; TJP, tight junction protein; TLR4, toll-like receptor 4; TRP, tryptophan; TYR, tyrosine; Dashed line in BBB: impaired BBB integrity; Dashed in in gut epithelial barrier: leaky gut.

The HPA axis starts its response by producing corticotropin-releasing hormone (CRH) in the hypothalamus, CRH then travels to the pituitary gland and stimulates the production of adrenocorticotropic hormone (ACTH) (17). ACTH subsequently travels to the adrenal gland via the blood and stimulates the production of glucocorticoids such as cortisol (**Figure 2**). Cortisol in turn inhibits its own production at both the pituitary gland and hypothalamus, creating a negative feedback loop.

Possible reasons for the involvement of stress and the HPA axis in depression could be the fact that chronic stress and stressors can result in a psychological downward spiral in humans, which can cause difficulty adjusting to continuously stressful situations, which in turn is related to exhaustion and ultimately depression (5, 35). The continuous exposure to stressors can for instance disturb receptor signaling in the amygdala, ultimately leading to the HPA axis activation (36). Moreover, it is thought that overactive cortisol production can lead to damage of the hippocampus and increase vulnerability to MDD.

### 4.4 Immune system and the pro-inflammatory state

Another consequence of stress and a dysregulated HPA axis can be suppression of immune function by affecting cytokines and cytokine production (30, 37). A recent comprehensive systematic review investigated the impact of MDD on 36 comorbid diseases found that the HPA axis was dysregulated, and the immune system was affected in these comorbidities (38). They also describe inflammation in general (whether induced by a disbalance in the HPA axis or not) as an underlying biological mechanism of MDD. This inflammation is related to elevated circulation of especially pro-inflammatory cytokines, for example TNF and IL-6, which are also associated with MDD (14, 39). Both hyperactivation of the HPA axis as well as immune activation during depressive episodes have been observed by other research (19). IL-6 (a marker of systemic inflammation) levels might even predict risk of the onset of MDD and poor antidepressant treatment response (40–42).

Pro-inflammatory cytokines can stimulate the HPA axis by binding to it (**Figure 2**) (15, 32, 43). This HPA axis hyperactivation can further increase the expression of cytokines, ultimately creating a positive feedback loop. Another important effect of pro-inflammatory cytokines and elevated stress is the increase in permeability of the blood-brain barrier (BBB) (**Figure 2**) (19). When BBB integrity is impaired (‘leaky BBB’), substances like pro-inflammatory cytokines can cross the BBB more easily, reaching the brain and affect mood and behavior (44). When pro-inflammatory cytokines disrupt the regulation of such mechanisms, the body can subsequently enter a state known as the ‘pro-inflammatory state’ (45, 46).

The pro-inflammatory state refers to a bodily condition characterized by malfunctioning of the immune system, indicated by elevated levels of proinflammatory cytokines (45). This systemic inflammation is seen as a physiological trigger of MDD (**Table 1**) (46). Research in humans showed that inflammatory factors are higher among depressed patients compared to controls. Studies found that inflammation can affect regional brain activity, neurogenesis and changes in microglia and astrocyte-specific markers in several brain regions (7). Moreover, pro-inflammatory drugs can induce depressive symptoms and increase the risk of MDD onset (47). Also in rodents, researchers found that systemic inflammation can cause long-term cognitive damage (48).

### 4.5 Gut microbiome in relation to MDD

While the HPA axis and pro-inflammatory state are significant factors, we believe there is another crucial player in the etiology of MDD related to the stress- and immune system: the gut microbiome (GM) (39, 49). The importance of the GM therein stems from its ability to exert an effect on many of the previously discussed concepts: the HPA axis (30), the immune system (50), the CNS (51) and the BBB (**Table 1**, and **Figure 2**) (19). Additionally, over 90% of serotonin is produced in the gut (19). These findings indicate an intriguing relationship between the microbiota in the gut, the brain, and the development of MDD, warranting a more comprehensive analysis.

The GM encompasses all microorganisms including bacteria, viruses, fungi, and archaea (52). However, because the majority of information is derived from bacterial studies, we will primarily focus on bacteria in this manuscript. It’s important for human health, metabolism, protection against toxins, pathogens, and cytokine secretion (53, 54). Moreover, the GM can impact cognition and emotions, partly by producing or modifying NTs and neuropeptides (54). Altogether, the GM is influenced by numerous factors like genetics, environment, diet, antibiotics, probiotics, and prebiotics (21, 45).

When the gut is disrupted, leading to an imbalance known as ‘dysbiosis,’ it may contribute to mental health disorders like MDD (**Table 1**) (49, 54). Factors like pathogenic bacteria, stress, antibiotics, and diet are associated with dysbiosis. This imbalance is linked to a higher risk of comorbid conditions between gastrointestinal diseases such as irritable bowel syndrome (IBS) (55) and obesity (56) and stress-related disorders such as depression (49, 54). Additionally, the GM is associated with neurological and psychiatric disorders like schizophrenia, autism, Parkinson’s disease, and multiple sclerosis (49).

The link between the brain and GM is known as the MGBA, a bidirectional connection involving the CNS, enteric nervous system, and digestive system (21). It plays a role in gut movement, hormone and NT secretion, HPA axis, immune system. Given its central role, we will conduct a more comprehensive analysis of the MGBA in the upcoming section.

## 5 MGBA and MDD

The MGBA has been thought to play an important role in neurological and psychiatric disorders such as Parkinson’s disease, Alzheimer’s disease, autism spectrum disorder and MDD (30). Various studies show that the bidirectional interaction between the GM and the brain affects CNS development and cognitive functions such as stress regulation, behavior and mood (**Figure 2**) (21, 30). The fact that these CNS functions and pathways are impaired in MDD supports the idea that the MGBA is involved in depression. Moreover, the significant impact of microbiota in neural plasticity and circuitry wiring during neurodevelopment could heighten the vulnerability to stress-induced psychiatric disorders such as MDD (16).

Developmental research demonstrated that the GM is able to influence postnatal development of the HPA response in mice (57). Furthermore, the GM directly influences the development of the brain, observed in germ-free (GF) mice that show abnormal microglia morphology, modified gene expression, and an impaired functional response to stimulation (50). Besides directly influencing the developing brain, the GM also influences the mature brain and neurons (58).

Animal research supporting this idea involved fecal microbiota transplantation (FMT), a technique where researchers transplant fecal matter of human patients with MDD and healthy controls into rats with a depleted gut microbiome. In the rats that received fecal matter originating from MDD patients, the transplantation led to more behavioral and physiological characteristics typically seen in depression, compared to the rats that received fecal matter from healthy controls (59). Also, GF murine models compared to their non-GF counterparts showed remarkable alterations in the brain, immune system, HPA axis, microglia and BBB, which are implicated in anxiety and MDD behavior (58, 60). The relation between GF rodents and MDD may be attributed to the fact that GF mice show morphological alterations of neural dendrites in the amygdala and hippocampus (16). Interestingly, external stressors like maternal separation in rodents also led to behavioral despair, alterations in the HPA axis and changes in gut commensals (21). However, due to the fact that these studies are conducted in animals, it is crucial to exercise caution when interpreting the results, as they serve as mere “depression models”.

In humans, research showed similarity in fecal microbiota signatures of patients with irritable bowel syndrome (IBS) and patients with MDD (55, 61). IBS is characterized by gut dysbiosis, including abdominal pain and bloating. Research found that the prevalence of depression in groups of people with IBS (38.7%) was significantly higher than the prevalence of depression in the control group (6.5%) (62). Moreover, relative to the control groups, depression scores (assessed using the Hospital Anxiety and Depression Index and beck depression inventory) were higher in the IBS groups (63). Concerning microbiota, research showed that the concentration of *Lactobacillus* in feces was lower for healthy students in a period of intense stress compared to a period of mild stress (21). Furthermore, a systematic review found that depressed patients had a 58% higher risk of becoming obese, and obese patients faced a 55% higher likelihood of experiencing symptoms of depression throughout their lives, showing the bidirectionality of depression and the MGBA (56).

Though it is important to realize that these correlations are not necessarily causal relationships, they give a clear indication of a link between MDD and diseases related to the MGBA. In order to utilize this knowledge to develop treatment possibilities, it is crucial to understand the process through which the connection between MGBA and MDD is established.

### 5.1 The interplay between the dysbiosis, the pro-inflammatory state and a dysregulated HPA axis in MDD

The contribution of the MGBA in the etiology of MDD roughly consists of the complex network of interactions between dysbiosis, the pro-inflammatory state and a dysregulated HPA axis as major players (21, 50). For instance, chronic stressors influence the GM composition, resulting in activation of the HPA axis and elevation of the pro-inflammatory state (19). As mentioned earlier in this review, dysregulation of these mechanisms are known to worsen symptoms of MDD (30, 47, 49). To create an overview of current knowledge, we visualized these concepts into a model, which can be found in **Figure 2**. Though this is a simplified representation, it gives an impression of the processes at play. The connections in the Figure will be clarified in the next sections.

In section 4.3, we explained the interaction between the HPA axis and the brain, which can be found at the top right of **Figure 2** (hypothalamus, pituitary gland, adrenal cortex, and biomolecules in between). As we discussed in section 4.4, the HPA axis is connected to the immune system, which is found at the middle left of **Figure 2** (immune cells). The immune system can lead to systemic inflammation and impair BBB integrity, affecting mood and the brain visualized at the top of the image (44). The BBB is displayed as the top box with a black border in **Figure 2**. What becomes clear from section 4.5, is that these processes are connected to the gut, which is visualized at the bottom of the image. The bottom box with the black border represents the gut epithelial barrier, schematically displaying the biomolecules and processes related to the MGBA inside. Another crucial structure in connecting the brain to the GM is the vagus nerve, visualized in the middle of **Figure 2** (64).

### 5.2 Vagus nerve

The vagus nerve (VN) is the tenth (X) cranial nerve which transmits afferent (sensory) and efferent (motor) sensory information towards and from the CNS to the periphery, forming a direct link between the brain and the gut (64). The VN communicates in a bi-directional relationship with the immune system and can be activated through short-chain fatty acids (SCFAs), and inflammatory processes in- and outside the periphery (65, 66). In other words, the VN forms a connection between the CNS and enteric nervous system mediated by immunoregulatory signals (19). The VN can affect appetite, mood, and sickness behavior, and possibly induce an immune response through efferent vagal signaling (65). Moreover, cytokine receptors that detect and react to inflammation are expressed on VN afferents. This in turn influences the activity of brain regions implicated in mood and motivation (60). Other research showed that vagotomy (removal of the VN) was associated with decreased neuronal activity and percentages of immune cells, and changes in gene expression and depression-related behavior in rodents (64, 67).

### 5.3 Microglia

Other cells involved in the MGBA-related etiology of MDD are microglia (**Figure 2**). Microglia are macrophage-like cells in the brain, functioning as important immune cells that detect changes in the environment. Microglia are also involved with neuroinflammatory processes and are a part of MGBA communication, therefore possibly involved in the etiology of MDD (45). The GM plays a critical role in multiple aspects of microglia including maturation, morphology, and immunological function (68).

Microglia produce cytokines in the brain, and alterations in microglia and cytokines can result in neuroinflammation and is likely fundamental in MDD (60). Those cytokines may affect MDD through influencing growth factors (like BDNF) and the production of toxic metabolites. Additionally, neuronal destruction and the production of neurotoxic compounds may be related to symptoms of MDD. Moreover, there is a link between stress and regulation of immune responses that affect microglia in the brain, which can lead to neuroinflammation (69, 70).

### 5.4 Biomolecules

For connecting the mechanisms (i.e., HPA axis, immune system, MGBA, VN) together, **Figure 2** shows several biomolecules (such as SCFAs and lipopolysaccharides; LPS) that play their own part in the MGBA. These are interconnected, and play a role in the dysregulated HPA axis, pro-inflammatory state and dysbiosis. How these biomolecules exert their effect and connect the mechanisms described above will be explained in the next sections.

#### 5.4.1 SCFAs

Low levels of SCFAs have been associated with depressive-like behavior, compared to high levels of SCFAs (**Figure 2**) (15, 19, 71). SCFAs belong to the major gut bacteria metabolites and can offer relevant benefits in terms of depression relief, anti-inflammatory effects, neuroprotection, regulating T-cell induction, and a good BBB permeability balance (15, 16). This improvement of BBB integrity by butyrate (a SCFA) has been associated with the upregulation of tight junction protein (TJP) expression, which are proteins in the brain restricting substances to move freely between the brain and blood (71). Dysregulation of TJP is related to impaired BBB integrity, exposing the CNS to damaging substances. However, due to the MGBA being highly interconnected and SCFAs not being the only microbial metabolites, the causal link between SCFAs and the increase of the BBB remains uncertain. Moreover, reduction of SCFA-producing bacteria play an important role in dysbiosis, gut mucosal inflammation and loss of intestinal barrier integrity (leaky gut) (16).

#### 5.4.2 LPS

Another important biomolecule involved in MGBA and MDD is LPS (21). Research suggests that LPS has the capability to trigger depressive-like behavior in animal models (72). LPS is a microbe-associated molecular pattern (MAMP) and a large constituent of gram-negative bacteria that binds to toll-like receptors (TLRs) located on immune cells (19, 73). MAMPs are microbial-derived products which can activate immune cells to promote the release of pro-inflammatory cytokines, which increases permeability of the intestinal barrier (‘leaky gut’) and the BBB, and influence CNS function and behavior (15). Additionally, when pro-inflammatory cytokines are able to cross the (damaged) BBB, they can interact with neurons which can lead to sickness behavior and MDD. The activation of TLRs can also activate the HPA axis, which may result in further increment of BBB permeability and gut-membrane-permeability, the latter associated with leaky gut (19, 45, 74).

#### 5.4.3 Leaky gut and impaired BBB integrity

A leaky gut can be the result of the gut epithelial barrier being damaged by dysbiosis and is displayed in **Figure 2** as a dashed line (15, 47). The leaky gut has been associated with MDD through the immune system (60), and gut permeability markers are associated with patients with recent suicide attempts (42). Also stress in rodents might increase the leaky gut (74). However, direct mechanistic evidence between dysbiosis and a leaky gut is limited (75). What research does suggest, is that a leaky gut increases unregulated translocation of microbes over the lamina propria (thin layer of connective tissue, such as in the gastrointestinal tract). This can lead to, for instance, the infiltration of immune cells into the brain (71). It has been hypothesized that this infiltration can be pathogenic in the CNS because of the destructive properties of these cells.

Leaky regions in the BBB, called circumventricular organs, allow molecules and cytokines to travel to the brain, are related to systemic inflammation, and may cause altered brain function (76). Immune cells for instance produce cytokines like IL-17A, which further impair BBB integrity (displayed in **Figure 2** as a dashed line) and contribute to neuroinflammation (77). This mechanism has been associated with CNS diseases such as multiple sclerosis and morbus Parkinson (78).

Moreover, a leaky gut allows LPS to activate even more TLRs inside and outside the gut. Because of increased BBB permeability and a leaky gut, LPS reaches systemic circulation and is therefore able to travel to the brain, where they can bind to TLR4 located on brain endothelial cells (cells which are part of the BBB), displayed at the top left of **Figure 2** (79). Here, LPS can alter TJP expression, contributing to impaired BBB integrity, immune cell trafficking, and the release of more pro-inflammatory cytokines (71). Interestingly, TLR4 has been found to be upregulated in MDD patients (14). Moreover, when MDD patients were successfully treated, the TLR4 levels were found to be restored, suggesting their potential role in depression.

This example highlights how the immune system, the gut, BBB integrity and MDD are interrelated. However, there are many more complex interactions like these involved, but covering them each individually is outside the scope of this review (60, 64).

#### 5.4.4 Neurotransmitters and other signaling molecules

On top of the biomolecules described in the previous section, neurotransmitters (NTs) are thought to play an important role in the MGBA (**Figure 2**). Gut microbiota are able to secrete multiple NTs (and precursors), neuropeptides and metabolites. The NTs that are released by different bacteria species are GABA, acetylcholine, serotonin, dopamine, and histamine (19, 20). Another study even suggests that various *Lactobacillus* spp. can synthesize all the above-mentioned NTs (32). From the gut bacteria, NT (precursors) can travel through the blood or the VN to the brain (80).

Synthesis of NTs and neuropeptides that regulate cognition and behavior is in turn partly modulated by SCFA (mainly butyric and propionic acid) (81, 82). They enhance tyrosine and tryptophan hydroxylase expression, which are involved in dopamine, noradrenaline, and serotonin synthesis (81, 82) and have neuroprotective properties (**Figure 2**) (83).

#### 5.4.5 Monoamines

Serotonin, dopamine, and norepinephrine are the monoamines that are mostly associated with MDD (7). All three are modulated by antidepressants such as SSRIs and noradrenaline and dopamine reuptake inhibitors. Serotonin is thought to be a crucial NT which is known as the primary regulator of mood and cognition (82, 84). Notably, 90%–95% of serotonin is compartmentalized in the gut, and serotonin production can be regulated by the GM (84). Another article mentions that patients with MDD generally have low circulating levels of tryptophan, possibly because low levels of plasma tryptophan are related to alterations in immune function (64, 76). As tryptophan goes predominantly through the kynurenine pathway, it is interesting that research showed that the kynurenine/tryptophan ratio was significantly higher in depressed individuals compared to healthy controls (59). Additionally, dopamine and noradrenaline are also NTs that have an influence on the CNS and are produced by microorganisms in the gut (**Figure 2**). For instance, stress in mice showed increased levels of dopamine and noradrenaline in the gut (85).

However, there is a major controversy about the association between MDD, serotonin and tryptophan. A recent paper involving 17 studies (systematic reviews, meta-analyses and more) concluded there is no consistent support for an association between serotonin and depression (10). Also, the relationship between tryptophan and serotonin remains weak. The weak relationship between MDD, dopamine and norepinephrine has not been investigated as comprehensively, but because of the controversy around serotonin, careful interpretation is required (10). This does not necessarily mean the monoamines are not involved in the MGBA, but the interaction is more complex and the relevance for MDD seems to be far less than previously assumed. However, it is important to reiterate that the study by Moncrieff et al. (10) has faced significant critiques regarding its reliability, emphasizing the complexity of serotonin’s role in MDD.

#### 5.4.6 Nitric oxide & oxidative stress

Another NT associated with microbiota influencing MDD is nitric oxide (NO) (**Figure 2**). The gastrointestinal tract is rich in sources of NO and the GM is known to be involved in oxidative stress (81). Nanomolar concentrations of NO seem to have a neuroprotective effect, but excessive NO production can be neurotoxic – associated with neuroinflammation, cellular damage, axon degradation, and neurodegenerative disorders including MDD. Excess production of NO may lead to the generation of reactive oxygen species and reactive nitrogen species, both causing oxidative stress. This can lead to cellular and DNA damage. Depressed patients show a significant increase in oxidative stress (47, 81). This may have to do with the fact that overproduction of reactive oxygen species characterizes activation of the inflammatory pathway. Also, research shows that endogenous antioxidants can be decreased in MDD patients (81, 86). This is in line with research that showed that depletion of the GM might also affect the function of antioxidants (87).

#### 5.4.7 Brain-derived neurotrophic factor

Alterations in brain-derived neurotrophic factor (BDNF) modulation are also a risk factor of MDD in which microbiota can play a role (**Figure 2**) (18). BDNF is a neurotrophin and growth factor that has neuroprotective effects, and plays an essential role in the survival of neurons (64). It is widely expressed throughout the CNS and especially active in the hippocampus (30, 64). Decreased levels of BDNF in the hippocampus are associated with depression and are often seen as comorbidity in IBS and other inflammatory-bowel diseases (84). Furthermore, various treatments for depression, such as antidepressants, show an increase of BDNF expression in the brain (64). BDNF has also been used as a marker for antidepressant effects (70).

It is important to stress that bacteria can be considered beneficial or non-beneficial, as indicated in **Figure 2**. For example, some bacteria species can promote cytokine production (54) and increase anxiety (64), while other bacteria can reduce anxiety-like behavior (4, 17, 43). In line with these results, some studies found an effect of probiotics on the HPA axis (88), while others did not (89). Also, antibiotics can exert both positive effects and negative effects on the GM and MDD (69, 87).

Taking the information of section 4 and 5, we suggest that MDD is not a brain-only phenomenon, but a gut-brain interaction phenomenon. Genetics and neurobiology certainly play a role, but are not the sole cause. The HPA axis and immune system are part of the interconnected MGBA, and dysregulation of the HPA axis, a pro-inflammatory state and dysbiosis contribute to the development of MDD. Additionally, the bidirectional connections seen in **Figure 2** regarding the HPA axis and GM, cytokines and the GM, cytokines and microglia and so forth highlight the overall bidirectional character of the MGBA. We believe that to treat depression more effectively, the MGBA is a crucial part to focus on and cannot be ignored.

## 6 Combining biomolecular mechanisms with a spiraling risk factor model

**Figure 3:**
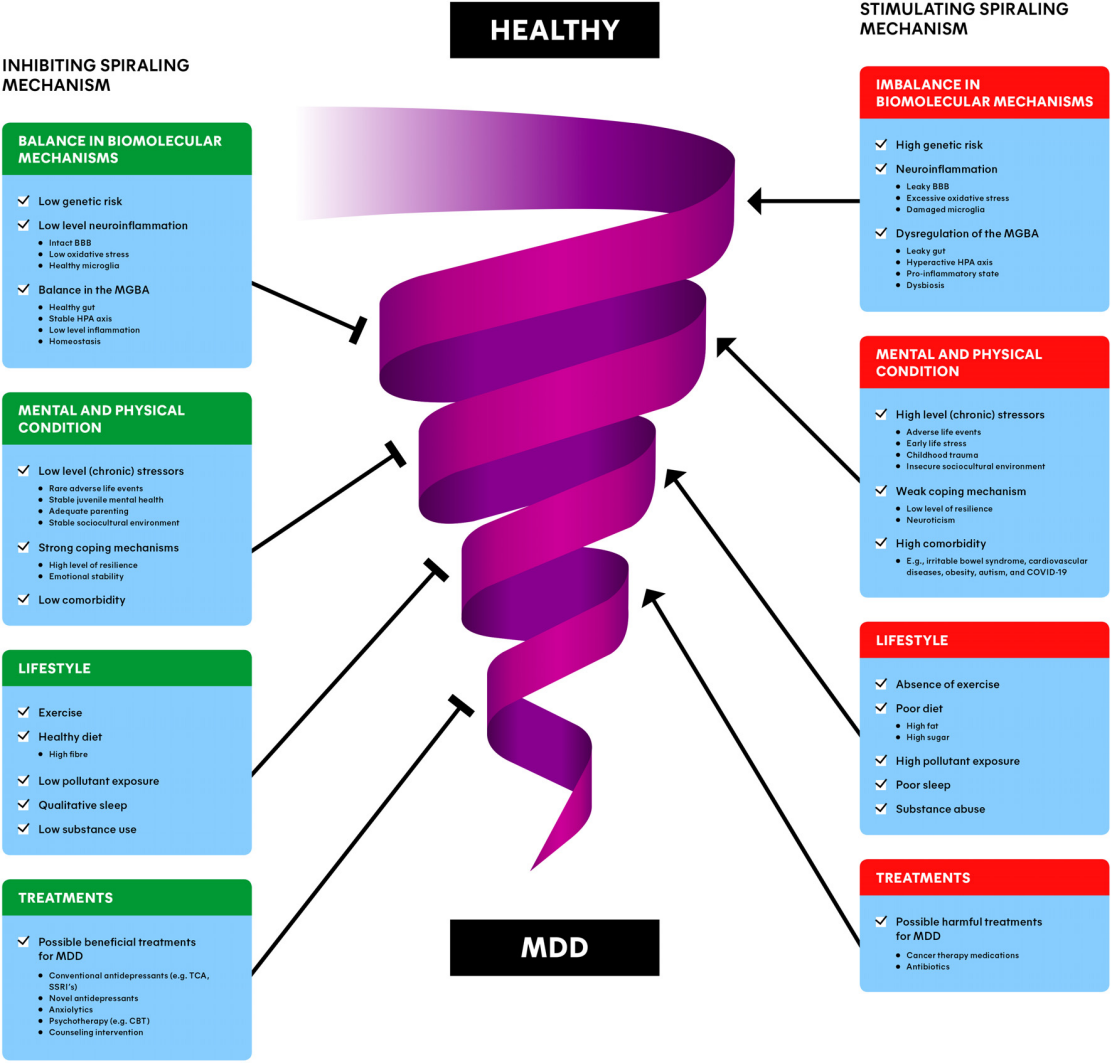
Spiraling risk factor model of major depressive disorder (MDD): inhibiting (left) and stimulating (right) risk factors interact dynamically, involved with the downward progression from a healthy individual to a depressed individual. The combination of balance in biomolecular mechanisms, the mental and physical condition, lifestyle and treatments of a patient/individual may provide the most promising and preventing interventions for MDD. BBB, blood-brain barrier; MGBA, microbiota-gut-brain axis; HPA axis, hypothalamic-pituitary-adrenal axis; MDD, major depressive disorder; TCA, tricyclic antidepressants; SSRI, selective serotonin reuptake inhibitor; CBT, cognitive behavioral therapy.

Although we believe the MGBA cannot be ignored in depression, MDD is a complex and heterogeneous disorder, in which psychological and environmental factors in addition to the biomolecular mechanisms contribute (5). This is why we propose a model that integrates the knowledge related to the biomolecular mechanisms underlying MDD with the psychological and environmental aspects (**Figure 3**). This spiraling model can be seen as the progression of an (im)balance in the condition of a person, divided in different groups of risk factors. Healthy individuals can obviously naturally encounter adverse life events and lead a less healthy lifestyle as well. This presents no problems while the balance is maintained. However, when a healthy individual slowly starts to experience a more depressed mood, all the factors in **Figure 3** can have an impact on the condition of the patient and eventually move towards MDD in the worst case (5). Moreover, many risk factors interact in a stochastic manner, and can therefore contribute to the increase of other risk factors. For example, when an individual is victim of domestic violence, it may cause stress-related issues later in life which can, for example, lead to substance abuse. Subsequently, this substance abuse can worsen stress-related issues, forming a positive feedback loop, spiraling towards the development of MDD.

The spiral in **Figure 3** represents this downward progression from a healthy individual to a MDD patient and vice versa. The involved factors often reside at opposing ends along a single dimension. For instance, an insecure sociocultural environment (e.g., unemployment or low income) is a risk factor, while a stable sociocultural environment (e.g., secure employment and high socioeconomic status) is a risk-reducing factor for depression (90). This is why each factor is depicted as a stimulating component (on the right) for the progression towards MDD, as well as an inhibiting component (on the left of the Figure), away from MDD. We differentiate between the balance in biomolecular mechanisms, the mental and physical condition, lifestyle, and (beneficial/harmful) treatments of individuals. Please note that the information described in section 4 and 5 is incorporated in the ‘Dysbiosis of the MGBA’ section. The upcoming sections are devoted to clarifying each of the groups of factors.

### 6.1 Balance in biomolecular mechanisms

The underlying biomolecular mechanisms are playing an important role in the etiology of MDD as described in section 4 and 5 (**Figure 3**). There are several genes (e.g., DRD2 and CLEF4) and epigenetics (e.g., histone deacetylases) associated with risk for developing MDD (7). Additionally, differences in brain regions like the hippocampus and the prefrontal cortex can play a role in depression. These risk factors can possibly disturb the balance of other biomolecular processes, or can be the result of other biomolecular imbalances, for instance related to the GM (**Table 1**) (7, 36).

A healthy individual with a low level of neuroinflammation naturally exhibits fluctuations in the levels of cytokines, stress-hormones and other biomolecules found in the gut (91, 92). This can become a problem when the homeostasis gets out of balance, for example because of MDD or comorbidities such as obesity and IBS (55, 61). Subsequently, the body can enter a state of dysbiosis (49, 54), pro-inflammatory state (45) or a hyperactive HPA axis (31), all signs of dysregulation of processes related to the MGBA (**Figure 2**). These states are associated with a leaky gut (60), impaired BBB integrity (77), disrupted levels of biomolecules (cytokines, cortisol, SCFA, LPS, monoamines, BDNF), oxidative stress (81), and impaired function of biological structures (VN, microglia) (18, 45, 64, 79) (**Figure 2**). Imbalances like these are in turn associated with neuroinflammation and MDD (**Figure 3**) (60, 69, 77). More detailed examples can be found in section 5.

### 6.2 Mental and physical condition

Zooming out from the biomolecular mechanisms, the mental and physical condition of a depressed patient play a major role in the spiraling mechanism. Stress is a consistent finding in MDD, and there are numerous comorbid diseases related to depression (5, 7). To overcome adverse life events, individuals must find ways to cope with them (93). Another large factor that plays a role in the current mental and physical state of individuals is to what extent people experienced juvenile mental health problems and trauma in their childhood (94). The impact of (chronic) stressors (including early-life stress and sociocultural determinants; SDs), comorbidity, coping and childhood problems will be clarified in the upcoming sections (**Figure 3**).

#### 6.2.1 (Chronic) stressors

Approximately 60–65% of MDD is explained by external factors such as adverse life events (5, 6). This is likely in large part the result of (chronic) stressors, as high levels of cortisol could potentially function as a predictive indicator of the risk of developing MDD (7). The source of stress could be in the past (e.g., early-life stress, childhood maltreatment or trauma) or it could be more recent (e.g., managing current life events and SDs) (**Figure 3**). On a psychological level, stressors can lead to avoiding, reducing, or predicting behavior towards a certain stressor (e.g., avoiding social interactions). From a biological perspective, stressors increase the level of cortisol of the individual, disrupting the HPA axis. Moreover, slightly higher cortisol levels in MDD patients have been found compared to controls (32, 34). Interestingly, MDD also increased cortisol levels in response to stressful stimuli. This fits the model as van der Gronde et al. (5) postulated: ‘depression is the result of a failure of coping mechanisms to control the stressors and a differential dysregulation in the stress system’.

#### 6.2.2 Early-life stress

According to a meta-analysis from 2019, people who went through early-life stress had higher odds of developing MDD prior to reaching the age of 18 years old compared to those who did not have a history of early-life stress (94) (**Figure 3**). They also found that the type of early-life stress plays a role in juvenile mental health. Poverty, illness/injury, and natural disasters were not associated with MDD, while emotional abuse and death of a family member were more strongly related to depression. Other than MDD, adverse childhood experiences are associated with significantly higher odds of anxiety, internalizing disorder, and suicidality in more extreme cases (95). Another large part of early-life stress in children and adolescents is negative behaviors of their parents (7). These negative behaviors could include hostile behavior and lower engagement. Additionally, depressed parents may also increase their children’s risk for developing depression.

Depression among children and adolescents is in turn associated with poor school attendance like absenteeism and truancy (96). These school performances may then again contribute to the downward depression spiral (**Figure 3**). Research trying to find potential underlying mechanisms demonstrate that resilience can partly protect against detrimental effects of child maltreatment (97). From a biomolecular perspective, research found altered HPA stress responses and lower levels of glucocorticoid receptor mRNA and other epigenetic differences in the hippocampus of humans who experienced childhood trauma (98).

#### 6.2.3 Sociocultural determinants

SDs are also essentially external sources of (chronic) stressors and can be associated with MDD (90) (**Figure 3**). SDs include economic security, social protection, recent positive events (e.g., holidays), equality and neighborhood safety (7). Generally, because SDs differ to such a large extent between individuals, it is challenging to determine whether MDD is a cause or consequence of SDs and rule out confounding factors.

A report by the world health organization (WHO) described that in every age and phase of life, challenges such as poverty, violence, inequality, and environmental deprivation pose a threat to mental health (99). Additionally, a recent comprehensive review demonstrates that depression is associated with failure to complete secondary school, unemployment, work disability, lower income-earnings and household income (90). Vice versa, employment has been shown to lower the likelihood of depression, potentially possibly by enhanced autonomy, socioeconomic status, and personal growth opportunities. Interestingly, however, better SD conditions do not mean beneficial effects only by definition, as another paper found that higher parental education correlates with increased prevalence of alcohol and drug usage during early adulthood (100).

From a biomolecular viewpoint, earlier work reported that lower socioeconomic status during adolescence was linked to epigenetics (101). Increased methylation, a chemical modification in DNA, of the serotonin transporter gene predicted heightened reactivity of threats of the amygdala, a brain region involved with processing fear. This amplified amygdala reactivity in turn moderated the connection between a positive family history of depression and the later development of depressive symptoms. If reproduced, this prospective pathway could serve as an innovative target biomarker for intervening and preventing mental health issues in individuals at high risk.

Counseling interventions, which provide guidance and support to enhance health and well-being, play an important role in the social environment of individuals with depression. This can be done by family or friends, or a professional such as a general practitioner (GP). Since friends and family typically do not monitor depression scores systematically, limited data are available on this aspect. What research does suggest is that adding cognitive behavioral counseling to standard depression treatments provided by GPs over six months is more effective in reducing depression symptoms and improving quality of life than usual care alone (102). Outside the GP, there is evidence that counseling provided by minimally trained community counselors effectively alleviated depression and anxiety levels (103).

#### 6.2.4 Coping mechanisms

Stressors are part of life, and healthy individuals naturally experience them (5). However, (mild) stressors may become a problem when they accumulate, become too much, and individuals fail to cope with high amounts of stress (**Figure 3**). This effect of chronic mild stressors accumulating and eventually contributing to the development of depression was also observed in rats (104).

Although the number of stressors matter, the ability of people to cope with these stressors is important as well (**Figure 3**). Resilience means the capacity to sustain or reclaim one’s psychological well-being in the midst of challenges (i.e., risk or threat) (7, 93). Research shows that it is evident that resilience is strongly related to mental health (93). This might be explained by the fact that people who are more vulnerable for stress (e.g., suffering from autism spectrum disorder) may develop MDD from mild stressors, while less vulnerable individuals may only experience MDD when exposed to severe stressors (5, 7). Research demonstrates that the level of resilience can vary from one person to another (93). This may differ between individuals based on genetics (105), but resilience is a multisystemic dynamic process that can develop through an individual’s life in which early-life stress can also have an effect (93). Additionally, a genome-wide association study demonstrated a strong positive genetic correlation between neuroticism (personal trait to react with negative emotions when confronted with threat, frustration, or loss) and MDD (106, 107) (**Figure 3**).

#### 6.2.5 Comorbidity

As mentioned earlier, MDD patients often share comorbid conditions along with their depression (5). A recent article mentions that approximately 75% of MDD patients will satisfy the criteria for at least one additional psychiatric disorder (22). This includes physical comorbidities such as cardiovascular disease, obesity and type 2 diabetes mellitus as well as mental disorders like anxiety, substance use disorder, psychosis, and autism spectrum disorder (5, 7) (**Figure 3**). Also, specific diseases like COVID-19 can be related to depression (108).

A recent comprehensive systematic review concluded that MDD was identified as a risk factor for both the development and exacerbation of various comorbidities (38). They assessed the association of MDD with about 36 comorbidities distributed over several groups including cancer, CNS, cardiovascular, metabolic diseases, autoimmune, musculoskeletal/pain, gastrointestinal, respiratory and substance use disorders. Based on the findings, autoimmune diseases and cancer were the least associated with MDD, while cardiovascular and metabolic conditions showed the strongest correlation (38). The study indicated that depression was more likely to increase the risk of developing comorbidities than existing comorbidities influencing MDD, except for substance use disorders. Interestingly, they discussed that a significant number of MDD-associated comorbidities are related to dysfunction of the HPA axis and the immune system, which supports our findings in sections 4 and 5. Moreover, numerous research studies acknowledge that, beyond biological mechanisms, the connection between depression and cardiovascular diseases could be influenced by diet and lifestyle factors (38).

Related to the MGBA, epidemiological data indicates that there is a positive correlation between obesity and an increased risk of developing mood disorders such as MDD (56, 109). As mentioned in section 5, a systematic review and meta-analysis of longitudinal studies found that depressed individuals have a 58% increased risk of developing obesity and that people with obesity were found to have a 55% increased risk of experiencing depression as time progressed (56).

### 6.3 Lifestyle

Besides comorbidities and dysregulated processes, research shows that the lifestyle of individuals plays a significant role in the etiology of MDD as well (110). This is an interesting topic, especially considering that individuals themselves can have a direct and non-invasive impact on lifestyle. Recent research focuses on lifestyle interventions to improve symptoms of depression (110, 111). Important lifestyle traits affecting the spiral of MDD are exercise, diet, sleep and substance abuse (**Figure 3**).

#### 6.3.1 Exercise

Research shows that individuals suffering from MDD exhibit decreased levels of physical activity and that these inactive people are at a higher risk of developing depression (110) (**Figure 3**). Aerobic exercise has even demonstrated comparable effectiveness to antidepressants, such as SSRIs like sertraline. The potential underlying mechanisms of exercise attenuating depression could be the stimulation of BDNF, enhanced sleep, the relief of stress, attenuated inflammation, increased activity in the prefrontal cortex and social factors like enhanced self-esteem and social interactions that help against loneliness (110).

However, while some meta-analyses support the potential antidepressant effect of exercise on MDD (112), other meta-analyses did not find significant effects (113). Nevertheless, another article states that exercise should be viewed as a supplementary component to complement other treatments for depression (8). Furthermore, exercise is associated with benefits to broader human health aside from depression, such as preventing and managing cardiovascular disease and obesity (114, 115). Together with the fact that exercise is a low-threshold intervention, exercise could be a favorable activity for people in general.

#### 6.3.2 Diet

Choices in diet can have a potential effect on the spiraling mechanism of MDD and might be related to the high prevalence of MDD in Western, urbanized countries (116) (**Figure 3**). A recent systematic review concluded that evidence suggests that a higher diet quality is related to an attenuated risk for the onset of MDD-related symptoms (117). However, another systematic review observed conflicting levels of evidence for different kinds of diets and depression (118). The fact that defining ‘a healthy diet’ is rather variable may contribute to these observed inconsistencies. Extreme diets may affect the risk of depression more severely. For example, individuals who consumed above half a liter of soft drinks every day had about 60% increased risk of having depression and depression-related symptoms compared to individuals not consuming soft drinks (119).

Research suggests that the GM plays an important role in linking diet and depression (120, 121). The ‘Western diet’ consists of highly processed foods that contain high amounts of fat and added dietary sugars (16, 47, 54). High sugar consumption is related to microbiota dysfunction, lower production of SCFAs, a pro-inflammatory state, a leaky gut and oxidative stress (47, 54). A high-fat diet for an extended period is recognized for its ability to induce chronic, systemic inflammation, increments of cytokines, and disrupt BBB integrity (44, 54). Furthermore, whereas high levels of non-digestible fibers promote the growth of beneficial bacteria (21), a lack of dietary fibers may be associated with higher depression scores (59).

#### 6.3.3 Pollutants

On top of diet, exposure to pollutants such as glyphosate, the most widely used herbicide globally, can play a role in the gut-brain axis and be a risk factor for depression (122). Glyphosate has been associated with increased mRNA expression levels of TNF and IL-6 (123, 124). This is particularly significant given that over half of the species in the central human gut microbiota are estimated to be sensitive to glyphosate (125). Related to pro-inflammatory cytokines, both microplastics and air pollutants have been associated with increased levels of TNF and IL-6 (126, 127) as well as depression (128, 129). Furthermore, heavy metals (e.g., lead and mercury) have been linked to dysbiosis and oxidative stress, which disrupts intestinal barrier permeability of the GM (130, 131). This is notable, as heavy metals have also been linked to depression (132).

#### 6.3.4 Substance abuse

For the last two decades, research already indicated the relationship between depression, and alcohol and drugs of abuse (133, 134) (**Figure 3**). Substance abuse can accompany depression as patients may seek to find a temporary escape to their depressed mood. However, alcohol and drugs of abuse only worsen depression in the long run due to their adverse effects (135). As alcohol and drugs have abuse potential, patients can become dependent on these substances.

A recent comprehensive systematic review containing meta-analyses found a fivefold risk between depression and cannabis dependence and a threefold risk between depression and substance use disorder (136). They even found a pooled odds ratio based on three studies of 11.3 for dysthymia (persistent depressive disorder) with drug dependence compared to no dependence. An older article described that about one-third of depressed patients also have a substance use disorder (134). Smoking is also related to depression, and cessation of smoking seems to improve psychological well-being as well (112). These results strongly support the idea that there is a consistent increased risk for comorbidity between depression and substance-related disorders.

Gut-brain interactions underlying this idea include the association between increased intestinal barrier permeability and substance abuse, particularly alcohol use disorder (137–139). Research showed that alcohol consumption is related to dysbiosis in both rodents (140) and humans (141), and that alcohol-dependent individuals show elevated levels of oxidative stress, TNF, LPS, IL-6, and systemic inflammation (137, 138). Also opioids, nicotine, and cannabis, have all been linked to changes in gut microbiota composition, highlighting its potential role in substance abuse (137).

#### 6.3.5 Sleep

Research suggests that sleep disturbances occur in 80-90% of depressed patients (7, 142). Indeed, a meta-analysis concluded that improving sleep was associated with significant medium sized effects on improving mental health (Hedges’ g = -0.53) and depression (Hedges’ g = -0.63) (143) (**Figure 3**). A possible contributing factor is the increase of usage of mobile phones. There is solid evidence that excessive mobile phone use, which is frequently reported, is correlated with the increased mental disorders such as depression, and poor sleep quality (144). This could be part of a lifestyle intervention to alleviate the symptoms of depression. Moreover, research shows that exercise interventions significantly improve sleep (145). On the contrary, sleep deprivation as a treatment has been associated with the improvement of depressive symptoms in certain subgroups of patients as well (146). However, this effect diminished after two weeks, was not superior compared to antidepressants, and the meta-analysis was based solely on post-treatment assessment. Nevertheless, sleep is an important factor to take into account in the spiraling mechanism (**Figure 3**).

### 6.4 Possible translation of the risk factor model to diagnosis, therapy and prevention

The next step is to use the spiraling risk factor model to suggest possible applications for treatment approaches against MDD (**Figure 3**). Three possible areas to examine are diagnosis, (multimodal) interventions, and prevention of depression.

#### 6.4.1 Early diagnosis

A possible promising application of our spiraling risk factor model including the MGBA is early diagnosis (**Figure 3**). Currently, MDD is diagnosed mainly based on the DSM-5-TR (American Psychiatric Association) and ICD-11 (WHO). Both assess depression based on behavior-related symptoms, in contrast to disorders such as cancer and diabetes, where diagnostic approaches have shifted towards genomic and other more objective tests (7). Although these-symptom based diagnosing methods are pragmatic, a subjective component remains present, as practitioners may assess MDD differently based on their own background, experience and culture. Moreover, details in the DSM and ICD change when a new version becomes available. It would be more objective to measure depression using biomarkers instead of behavioral symptoms, if validated biomarkers were available. Research might progress on this, considering high levels of cortisol and IL-6 may predict depression onset (7). IL-6 may be extra interesting, as gut permeability markers correlated significantly with IL-6 levels in depressed patients (42).

A current alternative diagnostic system that has made a start in this area is the Research Domain Criteria (RDoC) project by the National Institute of Mental Health early 2009. The aim of RDoC is to ‘develop, for research purposes, new ways of classifying mental disorders based on dimensions of observable behavior and neurobiological measures.’ (147). In this approach, neurodevelopment, environmental effects, cognition processes, and certain brain circuits are involved in the framework. However, critics have raised concerns about the uncertain validity of RDoC, and it seems there has not been much invested in the development of biomarkers yet (148).

Therefore, we believe it is worth investing in the research of biomarkers for MDD. A recent systematic review and meta-analysis concluded only cortisol may be a possible predictor for MDD, but high-quality and prospective studies are needed to underpin this hypothesis (149). Another paper concluded that changes in cortisol response variability is more associated with the development of MDD than absolute baseline levels for females (150). Thus, the predictive effect of cortisol may also be dependent on patient characteristics and subtypes of MDD, which research should take into account. Some research with immunometabolic markers, brain imaging and omics (i.e., genomics, metabolomics) has already commenced in this area (7).

#### 6.4.2 Multimodality interventions

For optimal results for moving patients up the MDD spiral, various treatment approaches should be considered (**Figure 3**). The current first levels of care for MDD are related to psychotherapies (7). Cognitive behavioral therapy (CBT) is the most validated and robust method, but other variants like supportive therapy and psycho-education are used as well (8). A newer development called digital therapeutics combines treatments like CBT with software-based (internet/mobile apps) technologies (151).

With mild-to-moderate MDD, psychotherapy is combined with a wide variety of pharmacological treatments including anxiolytics (5, 7). Conventional antidepressants are monoamine oxidase inhibitors (MAOIs) and tricyclic antidepressants (TCAs), but due to a more favorable benefit-risk ratio, they were mostly replaced by selective serotonin reuptake inhibitors (SSRIs) as first-line treatment for MDD (8). Interestingly, SSRIs might be associated with reducing anxiety and inflammation as well, highlighting the interconnected nature of the disease (152, 153). Besides the use of other monoamine reuptake inhibitors (e.g., selective norepinephrine reuptake inhibitors), anxiolytics (e.g., lorazepam), and novel antidepressant agents (e.g., bupropion, mirtazapine, ketamine and psychedelics), SSRIs remain the gold standard for pharmacological treatment of MDD (5, 8, 154).

For severe and treatment-resistant depression, somatic treatments such as electroconvulsive therapy (ECT) and transcranial magnetic stimulation (TMS) are used (7). The current most effective approach for treating resistant forms of depression involves a combination of pharmacotherapy, psychotherapy, and somatic therapies (8).

Besides established treatments, there are experimental MDD treatment options directly related to the MGBA. These include FMT and the use of prebiotics and probiotics (45), but there are limitations as well (155). While the probiotic *Lactobacillus rhamnosus* showed reduced stress behavior in mice, no significant effects related to stress-related measures, inflammation and cognitive performances were found in humans (156). Additionally, we do not believe that these ‘mono treatments’ are viable standalone treatments, as MDD is a heterogeneous disorder with a wide variety of psychological symptoms and environmental risk factors that extend beyond the MGBA (5) (**Figure 3**). MGBA-related treatments like FMT, prebiotics and probiotics may help correct the balance to some extent, but treating MDD effectively is likely to require multimodal treatment approaches including psychotherapy, pharmacotherapy and lifestyle interventions.

In addition to the prescribed treatments used to alleviate symptoms of depression, it is possible that an individual experiences effects of other treatments (recent or in the past) used for comorbid diseases. Discussing them all is outside the scope of this review, but we will cover some relevant examples.

Research shows that some patients treated with antibiotics show depressive complaints (87) (**Figure 3**). The link between antibiotics and MDD may be the result of antibiotics affecting not only pathogenic bacteria, but also commensal, protective bacteria. In addition, antibiotics can generate peripheral inflammatory factors which can cross the BBB or activate the HPA axis, increasing stress hormone levels. Contrary to this, other studies showed that antibiotics can also provide therapeutic benefits such as reducing neuroinflammation and depression-like behavior (69, 87, 157). The exact impact of antibiotics on MDD is likely to be complex and requires further study.

Immunotherapies to treat cancer, autoimmune diseases and allergies have been associated with MDD (158) (**Figure 3**). For instance, depression is the most common side effect observed in individuals undergoing extended treatment of the pro-inflammatory compound INF-α. Conversely, anti-inflammatory TNF antagonists like infliximab have the potential to alleviate depressive symptoms in individuals with inflammatory disorders, such as Crohn’s disease and ankylosing spondylitis (159). This shows the impact of the immune system on MDD, and could lead to the development of therapeutic options.

#### 6.4.3 Lifestyle interventions

An advantage of lifestyle interventions is that they are easily accessible and non-invasive for patients. This makes them accessible to combine with other treatment strategies if patients are willing to adhere to them. For example, research on dietary coaching demonstrated reduced depressive symptoms in patients with depression and improved prevention of MDD in healthy individuals (160). Moreover, although not all results are significant by itself, adding exercise is unlikely to be harmful and might help rebalance the spiral of MDD (110) (**Figure 3**). When someone abuses drugs, addiction facilities could be considered. As poor sleep is commonly found in many depressed patients, interventions to improve sleep patterns by a professional may help (142). Together, these separate interventions combined (involved with the brain, the gut and stressors) may move an individual significantly up in the spiral (**Figure 3**). Moreover, as limited access to treatment in low-income countries is a big problem, lifestyle interventions may be relatively affordable to implement compared to conventional treatments of MDD.

As lifestyle interventions alone are not likely to be sufficient as a standalone treatments, other aspects of the spiraling risk factor model need to be considered (**Figure 3**). Moreover, not all lifestyle interventions (exercise for example) provide consistent results (7, 113). This might have to do with the fact that patients often struggle with adherence to lifestyle interventions such as diet, exercise, or drug abuse reduction, limiting the results (161). Therefore, addressing the other factors in our spiraling risk factor model remains valuable.

#### 6.4.4 Prevention

Better than diagnosing and treating MDD is preventing the development of MDD. What becomes clear from **Figure 3** is that patients can move (slowly) from a healthy to a depressed state. If practitioners can intervene earlier or even prevent the initiation of the spiral altogether, it might be more efficient than treating patients where the interaction between multiple risk factors are ongoing (**Figure 3**). Prevention of MDD can be divided into three types: universal prevention (addressing the entire population), selective prevention (addressing high-risk individuals such as individuals who have experienced trauma recently) and indicated prevention (addressing individuals with subthreshold symptoms) (90).

Universal prevention is a society-level problem, as 85% of MDD patients have no access to treatment in low-income countries (7). Moreover, decreasing poverty and violence would help against MDD through several pathways in **Figure 3** (e.g., stable sociocultural environment, rarer adverse life events), but solutions for this are beyond the scope of this review. Selective prevention may be achieved by screening patients at high risk for MDD (e.g., soldiers returning from war, or those who experienced childhood trauma), or a school setting. Research of prevention in school settings involving behavioral and cognitive programs provided small overall effects (162, 163). This preventive effort allows for early intervention on pathways such as coping mechanisms or in more extreme cases (pharmacological) treatments (**Figure 3**). Indicated prevention has the potential to prevent patients who have some symptoms, but not enough to be diagnosed with MDD, from developing further symptoms. Lifestyle interventions (e.g., exercise and healthy diet) and evaluating the mental and physical condition (e.g., stressors and comorbidities) described in earlier sections might be more successful in an early stage and prevent further disease burden, inhibiting the downward spiral (**Figure 3**).

In addition to new early diagnostic and treatment approaches, multimodal prevention interventions should focus on exhaustion and sources of stress (5). Using a combination of psychotherapeutic treatment modalities could prevent the onset of MDD by improving the pace of learning new coping behaviors, exerting a synergistic impact on the developmental perspective, and breaking the downward spiral of stress and exhaustion. This might also help for other related mental disorders where exhaustion and stress are central, such as autism spectrum disorder.

## 7 Discussion

For the first aim of this manuscript (sections 4 and 5), we conducted a systematic review to investigate the biomolecular mechanisms and the role of the MGBA in the etiology of MDD. We proposed a schematic model to overview these processes (**Figure 2**). The second aim (section 6) was to integrate this knowledge with the psychological and environmental aspects of MDD, resulting in a comprehensive ‘spiraling risk factor model’ (**Figure 3**). Together, these models form an integrated biopsychosocial translational research framework for the prevention and treatment of MDD.

Our findings indicate that, in addition to genetics and neurobiology, the HPA axis, immune system, and gut microbiota are crucial biological mechanisms underlying MDD (**Table 1**) (21, 30). Imbalances such as HPA axis dysregulation, a pro-inflammatory state, and MGBA dysbiosis are interrelated and collectively impact the integrity of the BBB and intestinal barrier, contributing to the development of MDD (**Figure 2**). However, it is important to note that using biomarkers like LPS or SCFAs for diagnosing MDD is challenging due to the heterogeneous nature of depression, unlike conditions such as hyperthyroidism where thyroid-stimulating hormone levels serve as reliable indicators (164). Moreover, our review does not encompass all possible biomolecular mechanisms due to the scope limitations of this manuscript. Despite these limitations, our findings emphasize that MDD is a gut-brain phenomenon, which contrasts with the traditionally dominant serotonin hypothesis (10).

A limitation of our MGBA model is that dysbiosis, HPA axis dysregulation, and inflammation are not exclusive to MDD; they are also associated with other mental health conditions like anxiety and obesity (56, 64). Conversely, imbalances in the MGBA alone do not fully explain MDD, as psychological and environmental factors are also significant. We categorized these factors into mental and physical conditions (e.g., stressors, coping mechanisms, comorbidities), lifestyle factors (e.g., exercise, diet, substance use), and treatments (either beneficial or harmful for MDD) (5, 7). Together with the impact of the MGBA, we developed a spiraling risk factor model of MDD (**Figure 3**). Although we believe this model accurately represents the etiology of MDD, alternative configurations are possible. For instance, another study created a comprehensive flowchart focusing on the prediction of depression in male patients (165). Our model suggests that severe imbalances in one or more factors can lead to a downward spiral, resulting in MDD. This insight supports our integrated biopsychosocial approach as a viable translational framework for further clinical research on MDD.

Four potential focus areas for applying our translational research framework include early diagnosis, multimodal interventions, lifestyle interventions, and prevention. The RDoC initiative by the National Institute of Mental Health, which aims to develop new approaches for categorizing mental disorders based on dimensions of observable behavior and neurobiological indicators, aligns well with our framework (147). However, more research is needed on MDD triggers and biomarkers (7, 90).

A significant limitation of our manuscript is the lack of clinical evidence. Our ideas and models need to be tested in clinical research involving human subjects. Additionally, determining the prioritization of treatments is challenging due to the broad range of risk factors. Objectively testing whether our approach offers significant improvements over current treatment models is difficult given the heterogeneous clinical presentation of MDD. Although combined approaches are already in use to some extent, and current treatments are effective for many patients, our comprehensive approach should be considered primarily for patients who do not respond well to existing treatments or as a preventive measure (7, 8).

Research on MDD is inherently complex due to its heterogeneous nature and primary diagnosis based on behavior, which adds variability to the literature. Much of the research for our first aim is based on animal studies, which, while informative, require cautious interpretation when translating findings to humans (156). Nonetheless, there are advancements that correlate data from depressed patients with the gut-brain axis, linking gut microbiota characteristics with quality of life and depression (166).

Methodological limitations in our systematic review (sections 4 and 5) include potential selection bias from narrowing our search queries, which could reduce the sensitivity of our search strategy. Our goal was to gather a wide variety of studies, and broadening our search query helped maximize different perspectives. We did not systematically review study quality due to the diversity of studies, but content quality was reviewed collaboratively by the authors, excluding poor-quality studies. To enhance transparency, we preregistered our study protocol at PROSPERO.

The narrative review (section 6) is not a systematic review, requiring caution in interpreting the data. Literature sources were chosen through expert searches, possibly overlooking contradictory studies. This manuscript aims to offer new perspectives and directions for a biopsychosocial translational research framework for the prevention and treatment of MDD.

## 8 Conclusion

Our review indicates that the underlying biological mechanisms of MDD extend beyond genetics and neurobiology to include a dysregulated HPA axis, a proinflammatory state, and gut dysbiosis. These interconnected mechanisms are components of the MGBA, involving key biomolecules such as SCFAs, LPS, cortisol, NTs, BDNF, and IL-6, as well as structures like the vagus nerve and microglia. Imbalances within the MGBA contribute to impaired BBB integrity and a leaky gut, leading to neuroinflammation and the development of MDD. This suggests that MDD should be viewed as a gut-brain phenomenon rather than a brain-only disorder.

To incorporate the psychological and environmental aspects of MDD, our spiraling risk factor model considers the influence of mental and physical conditions (e.g., stressors, coping mechanisms, comorbidities), lifestyle factors (e.g., exercise, diet, substance use), and concurrent treatments for other conditions (which can act as either triggers or inhibitors of MDD). Clinically relevant imbalances among these various risk factors can worsen the condition of patients prone to MDD, driving them into a downward spiral.

Recognizing this interconnectedness, our biopsychosocial translational research framework emphasizes individualized, multimodal treatment strategies that address the whole system rather than isolated components like the brain, gut, or stressors. By integrating lifestyle interventions with existing therapies for MDD, we aim to more effectively interrupt the downward spiral compared to conventional treatments. Furthermore, MGBA-related biomarkers could enable a shift from symptom-based diagnosis and treatment to more precise, individualized care. Preventive measures targeting these biomarkers, along with stress- and anxiety-related triggers, could help alleviate the burden of MDD by facilitating interventions during earlier stages of the condition.

In conclusion, adopting a more integrated biopsychosocial approach to the prevention, diagnosis, and treatment of depression—integrating mental and physical health, lifestyle factors, alternative therapies, the MGBA, and symptomatic burden—holds the potential to significantly enhance outcomes for patients with MDD.

## Data Availability

The original contributions presented in the study are included in the article/**Supplementary Material**. Further inquiries can be directed to the corresponding author.
